# Politics and Physic

**Published:** 1856-03

**Authors:** 


### POLITICS AND PHYSIC.

It is a very difficult matter to prevent a politician from becoming a drunkard, and very few there are, who can run the dangerous gauntlet, without becoming lovers of liquor, at least to the extent of an occasional glass. The large number of distinguished political names which have passed down into a drunkard's grave within the last twenty-five years, will appal any one who will take the trouble to make the enumeration; and still more appalling would be the array of splendid minds, splendid in promise, whose glory has gone prematurely out, drowned in the wine-cup!

But the idea to which we wish to draw parental attention, in this article, is not to professed politicians, but to that numerous class of young men, who depend on political party for a living; in a large number of cases, their destination is one of three.

1. Premature death.
2. Brandy drinking.
3. A blank life.

It is well known, that most governmental employeés hold their position by reason of their political opinions, consequently, every change of policy throws them out of employment. Those who are not dismissed by an incoming administration, are such as have rendered their services necessary to the government, by their self-sacrificing assiduity in the faithful discharge of their duties; if this were all, it might be borne, but, as might be expected, a mere partisan office-holder neglects his duties, and the performance of them falls on those who are more faithful to their trusts, and in this double work, numbers perish prematurely, by diseases engendered through over-labor and over-solicitude.

But nine out of ten, of those who hold political places, change with the Administration, and being thrown out of office, have no other means of livelihood. With perhaps a wife and a child or two to be provided for, it is not difficult to perceive the weighty inducements such have to labor for another turn of the political wheel, and in performing that labor, they fall into such practices and associations, as make an escape from drunkenness an exception, rather than the rule.

But in the few cases where the love of liquor is not a result, where there is too much moral rectitude to go down to that degradation, the want of employment soon brings want of subsistence; then come despondency, idle habits, want of energy, and in its train want of ambition, and finally loss of self-respect and a "Blank Life."

In view of these things, we consider it a great calamity for a young man to obtain any salaried political office; better a great deal, because safer and immeasurably more independent, to serve a regular apprenticeship to some useful handicraft; for then, however bright may be the fortunes of after life, there will be in reserve, in case of reverses, a capital to draw upon, which misfortune can not sink, which governmental changes cannot destroy.

We feel safe in going still further, and recommend to every parent who reads this Journal, to sedulously avoid placing a child in any fixed salaried position, for such a position will engender habits of idleness, of inattention, of want of thoroughness, which will be an effectual barrier against success in life. A young man, with a fixed salary, soon begins to reason thus: "I will get so much any how, even if I am not quite so particular," and that is the first step towards doing things slightingly, and when such a disposition takes possession of any youth, he is virtually lost to society; for such a person will never obtain an enviable pre-eminence. Nor is this all, a fixed salary presents a direct bribe to laziness, it discourages activity and enterprise; for as to the odds and ends of time, which necessarily fall to persons employed to do business, the young man reasons thus: "If I do more than is required of me, if I work ever so hard, I get no more for it;" hence the time which now and then falls on his hands, is frittered away in some unremunerative manner, if indeed it is not spent in ways which ultimately end in a snare.

The point which we wish most to impress on parents is this: if you place your child in any salaried position, if you wish to encourage him, to stimulate his ambition, if you wish to inculcate a feeling of self-appreciation and self-reliance, which are absolutely essential to high success in any department of human life, place your children in positions which will moderately remunerate them, in proportion to their industry; we say "moderately remunerate," for we believe, that greatly disproportioned remuneration has dangerous and ruinous tendencies in more ways than one, for it engenders a taste for "short cuts" to wealth and that begets necessarily hazards, wasting anxieties, and desperate "throws;" then comes unscrupulousness, loss of principle, and with it loss of all that is dear to a business man. On the other hand if young persons are schooled to expect but moderate remuneration for their labor, that begets moderate desires, moderate ambitions, moderate expectations, and such only are the safe citizens in any community.

In conclusion, we desire to say, if a parent could only see one sight in a hundred of what any eminent city Physician witnesses of the foul and festering disease, of the bloated brutality which riots in the young, whom idleness or want of employment has ruined, they would feel relief in laying their children in an early grave, rather than see them placed in offices, however honorable and remunerative, the loss of which is so often attended with results already described.

We cannot but consider the general tendency, becoming still more common, to bring up children without mechanical employments, and without regular and thorough agricultural training, as one of the serious mistakes of the times, for not only must we become effeminate without labor, but that effeminacy is perpetuated in the offspring, while all of us must acknowledge that the hardy artificer and the sturdy farmer are the main elements of national thrift and national perpetuity.

